# Supplementary material for: Photonic machine learning implementation for signal recovery in optical communications
Source: Sci Rep. 2018 May 31;8:8487. doi: 10.1038/s41598-018-26927-y (PMC5981473; doi:10.1038/s41598-018-26927-y)
Supplement: Supplementary file 1 — Supplementary Material [file 41598_2018_26927_MOESM1_ESM.docx]

**Photonic machine learning implementation for signal recovery in optical communications – Supplementary Material**

**Apostolos Argyris*^,1^, Julián Bueno^1^ & Ingo Fischer^1^**

**^1^ Instituto de Física Interdisciplinar y Sistemas Complejos IFISC (CSIC-UIB), Campus UIB, 07122, Palma de Mallorca, Spain**

*** email: apostolos@ifisc.uib-csic.es**

**Supplementary Figures**


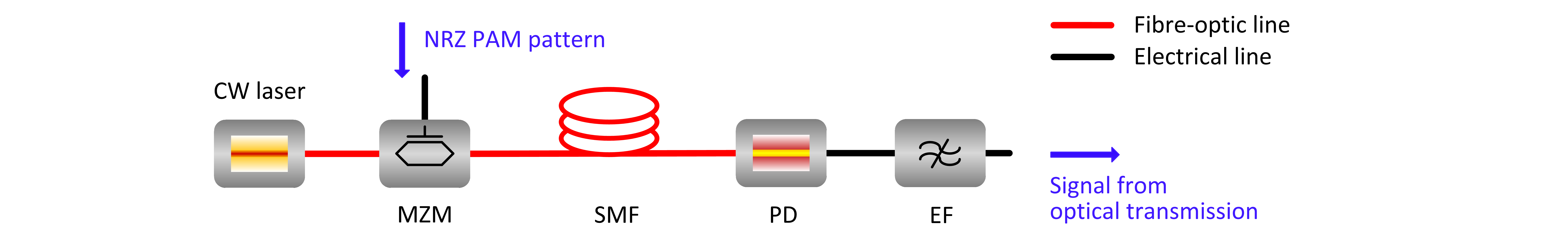


**Supplementary Fig. 1. Simulated topology of a short-reach transmission system.** A continuous-wave SL, with relative intensity noise (RIN) of -145dB/Hz, emits 10mW of optical power and is externally modulated by a Mach-Zehnder modulator with 30dB dynamical range. The modulation signal is a random PAM, NRZ pattern at *R_1_*=25Gb/s. A typical ITU-T G.652 SSMF is considered for transmission (*z_1_*), while the optical signal is detected by a PIN photodetector. A 4^th^ order Butterworth electrical filter with 20GHz bandwidth (0.8·*R_1_*) is used to filter out high frequency signal components.


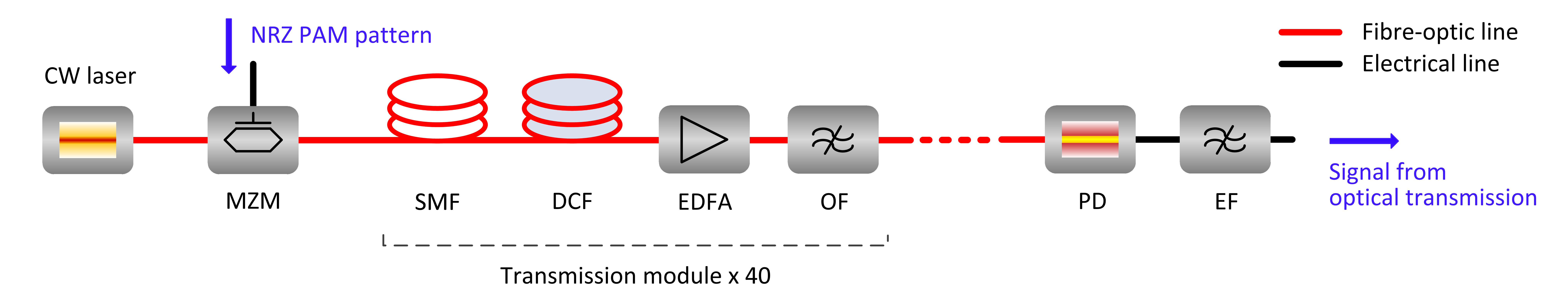


**Supplementary Fig. 2. Simulated topology of a long-haul transmission system.** For this system, the same SL emitter, MZM and modulation pattern are used, but the bit rate of the data stream is now *R_2_*=10Gb/s. The transmission line consists of 40 serially-repeated identical transmission modules. Each module consists of 100km-long SSMF, an appropriate length of dispersion compensation fibre that is matched to cancel completely the chromatic dispersion effects, an erbium doped fibre amplifier (EDFA) that compensates the power loss of the transmission path and an optical filter with *4∙R_2_* optical bandwidth. In our considerations, the total length of SSMF transmission is *z_2_*=4000km, without including the length of DCF. The detection stage is the same as in the configuration of the short-reach transmission system, while the electrical filter bandwidth is now reduced to 8GHz (0.8·*R_2_*).


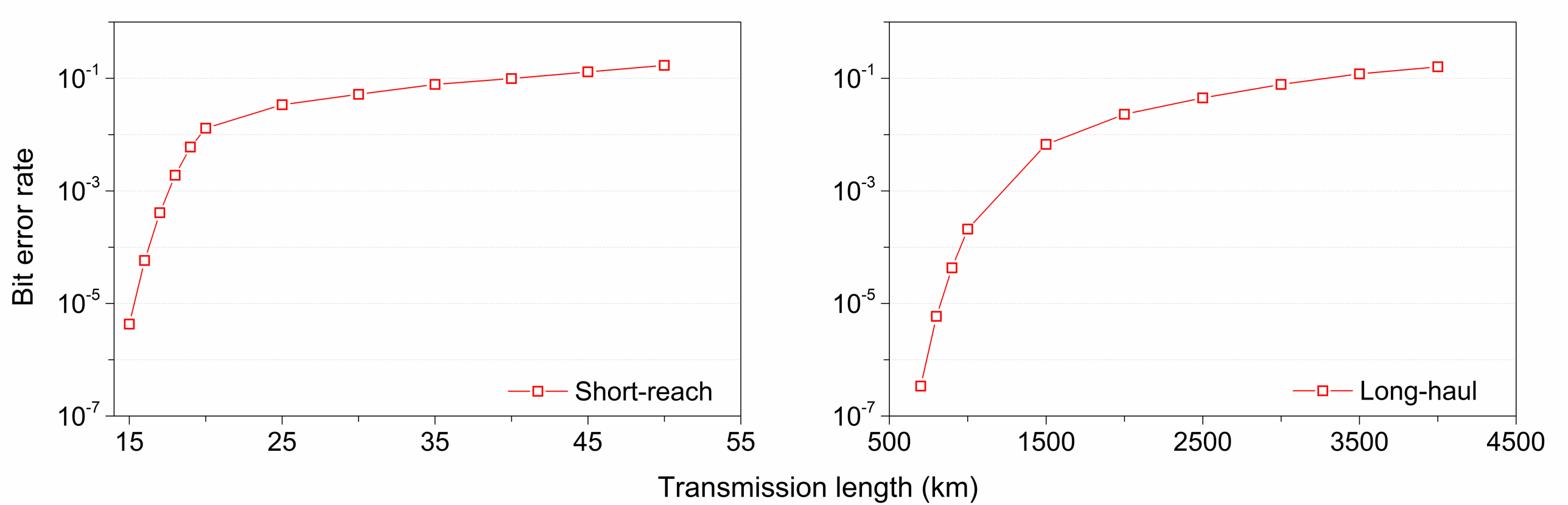


**Supplementary Fig. 3. BER versus transmission length for shot-reach and long-haul transmission systems.** For the transmission configurations presented in Supplementary Figs. 1 and 2, we estimate numerically the BER performance by considering different transmission lengths in SSMF. For the case of long-haul transmission we consider different number of transmission modules, by preserving the same internal structure. The BER of the decoded signal is higher than 0.1, for *z_1_*>41km in the case of short-reach transmission and for *z_2_*>3300km in the case of long-haul transmission. This consideration does not include any signal equalization with post-processing techniques.


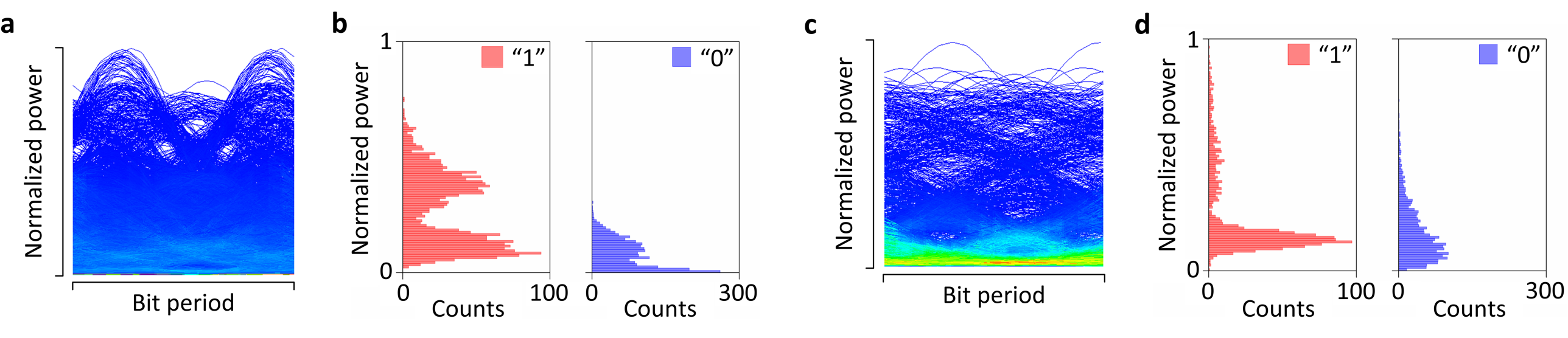


**Supplementary Fig. 4. Properties of the photodetected signals after transmission. (a)** Eye-diagram and **(b)** histogram of the detected power of the bit stream sampled at 0.6 of the bit period, for the short-reach transmission system at *z_1_* =45km distance. Chromatic dispersion and Kerr nonlinearity eliminate the potential for an efficient binary level separability. **(c)** Eye-diagram and **(d)** histogram of the detected power of the bit stream sampled at 0.6 of the bit period, for the long-haul transmission system at *z_2_* =4000km distance. Kerr nonlinearity and spontaneous emission noise from amplification units eliminate the potential for an efficient binary level separability.


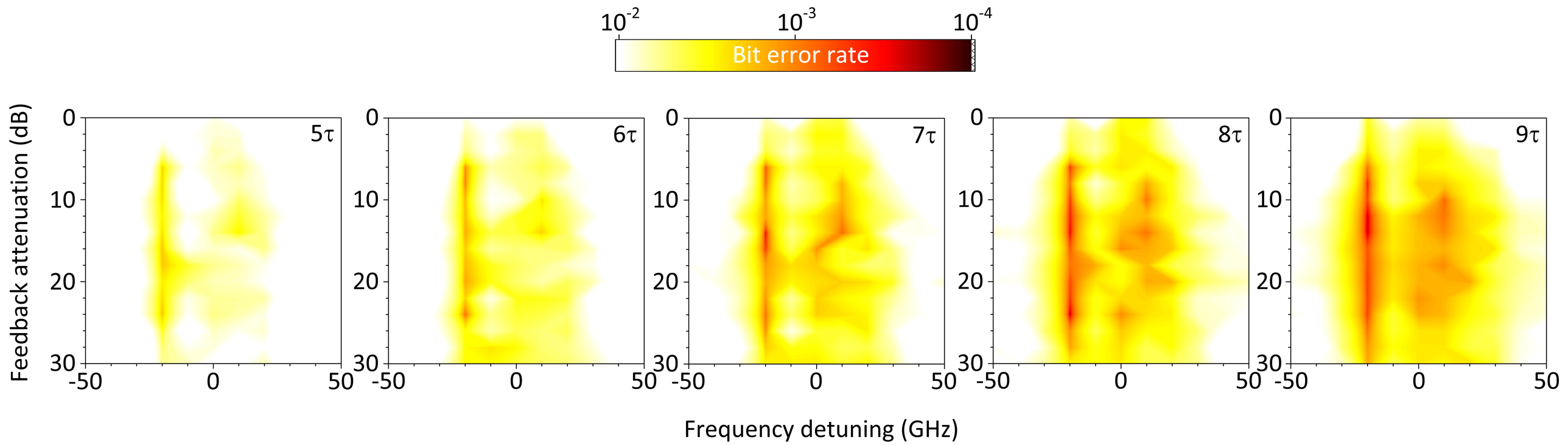


**Supplementary Fig. 5. Experimental BER mapping of the short-reach system after training on the reservoir output.** The mapping is performed versus frequency detuning *Δf* between the injection and the reservoir laser, while the feedback strength is controlled by attenuating the feedback that is available at the reservoir. Zero attenuation means that the feedback strength is defined by the setup structure and in our case this is roughly 20% (7dB less) of the emitted optical power of the reservoir SL. Moreover, the mapping is repeated for various numbers of bit timeframes taken into account for the linear classification (from 5*τ* to 9*τ*). In the short-reach transmission case, bit information is shown to spread up to 4 neighbouring bits. Each map is the average of the BER values taken from 5 independent measurements. The transient states of the photonic reservoir depend directly on its operating conditions and the injected signal. In our classification task, their properties determine the BER performance of the recovered signal. Complete injection locking conditions that appear around *Δf=-10GHz* for a wide range of feedback values, result in poor BER improvement. Completely unlocked conditions, observed for frequency detuning values below *Δf =-30GHz* and above *Δf =30GHz*, also result in poor performance. However, for partial locking regimes and moderate feedback conditions the system exhibits significant BER improvement. This improvement is observed when considering at least a 5*τ* timeframes response from the reservoir and is maximized for 9*τ*. The optimized BER is measured to be 1.8∙10^-4^ and is recorded for *Δf=-20GHz* and an excess feedback attenuation of 12dB. By minimizing the effect of feedback (30dB excess attenuation) we still get improved classification (BER=7∙10^-4^) compared with the benchmark tests. In this case the reservoir is degenerated to an ELM that benefits from the nonlinear transformation of the injected input to the semiconductor laser.


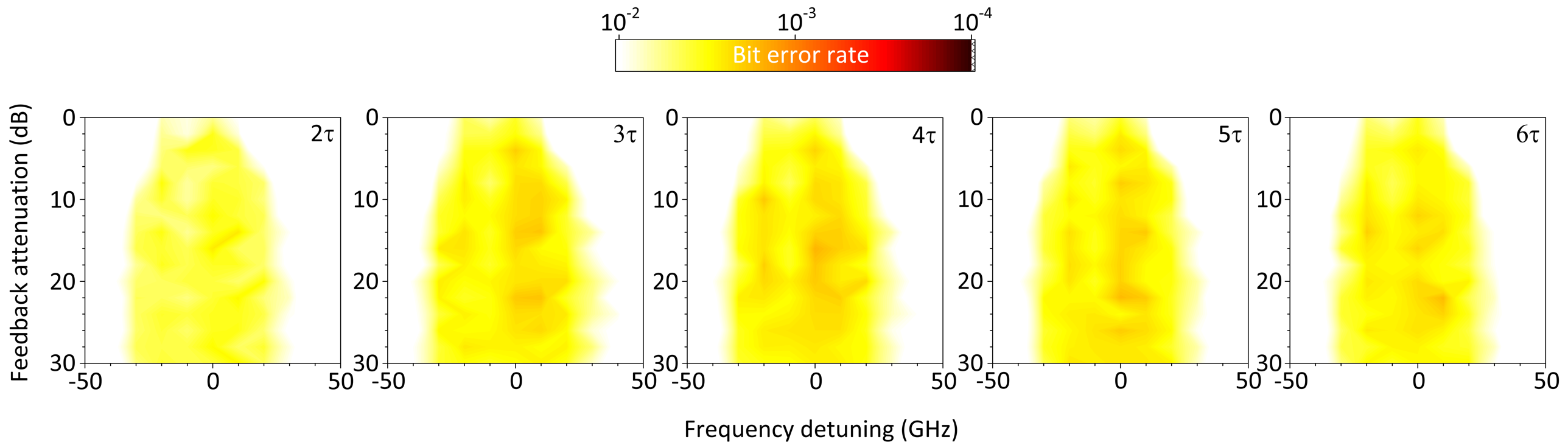


**Supplementary Fig. 6. Experimental BER mapping of the long-haul system after training on the reservoir output.** An analogous mapping with the one presented in Supplementary Fig. 4 is performed for the long-haul transmission case. In this case, the mapping is repeated for training with shorter bit timeframes (from 2*τ* to 6*τ*), since bit information spreads only to just one or two neighbouring bits, due to the dispersion compensation of the signal along the transmission line. Thus, the consideration of more virtual node responses from extended bit time-frames does not provide any additional information for the classification. Consistent observations regarding the locking conditions are also made here. Again, complete injection locking conditions that appear around *Δf=-10GHz* for a wide range of feedback values, result in poor BER improvement. We find the optimal operating condition at *Δf=0GHz* and at an excess feedback attenuation of 16dB, when considering a 4* *timeframes response from the reservoir; the measured BER is 1.7∙10^-3^.


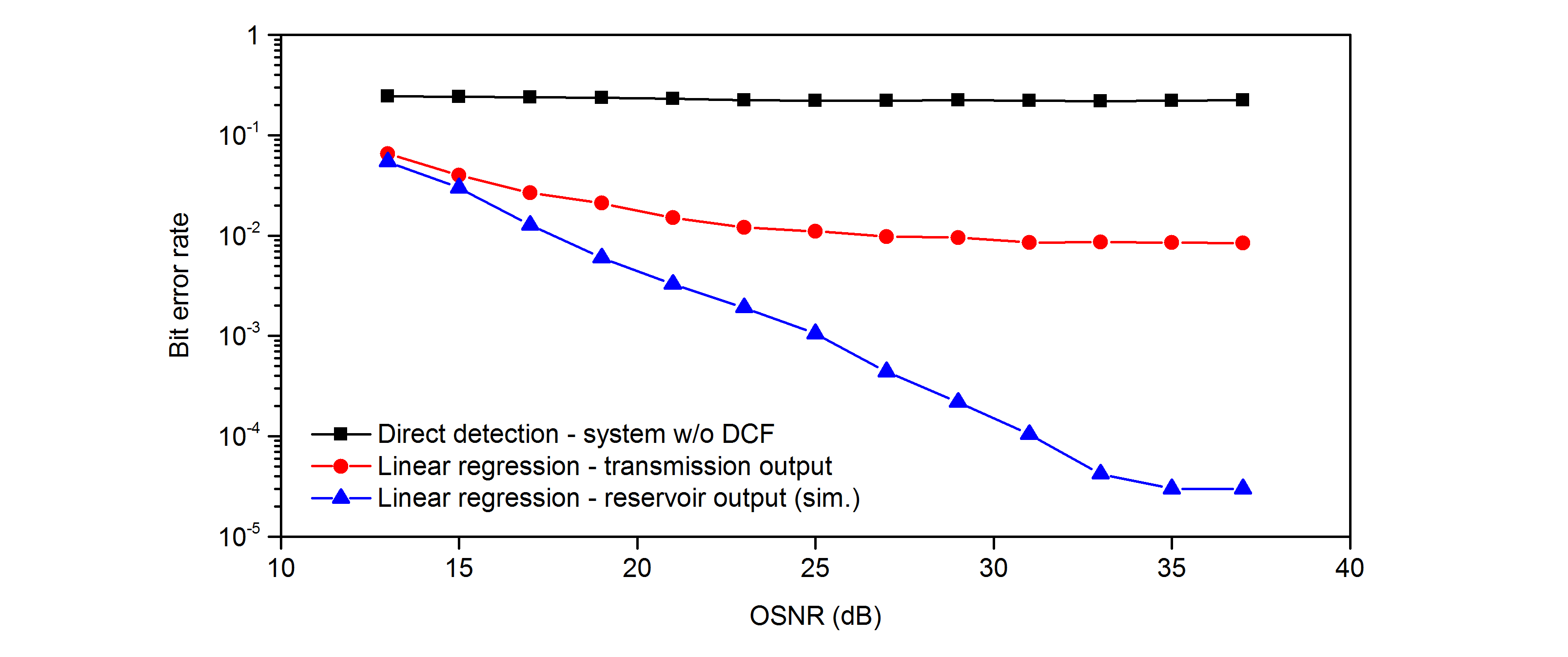


**Supplementary Fig. 7.** Bit error rate dependence on the optical SNR of the received signal in a short-reach communication system with *z_1_´*=50km. The reservoir properties that have been considered are: * *=1.6ns, *k*=32, *j*=4, *Δf*=5GHz and *k_f_=*0.05. This corresponds to a BER value <10^-4^, as shown in Fig. 5 of the manuscript. The dependence on the optical SNR is obtained by adding optical white noise before photodetection. We compare the BER performance, when considering a linear classifier with 9-bits of training (red dots) of the transmitted signal and the same classifier with 9-bits of training (blue triangles) of the photonic reservoir output. In both cases the launched optical power for transmission is 10mW. The analogous transmission system with direct detection without dispersion compensation is shown as a reference (black rectangles), it suffers from linear and nonlinear distortion and exhibits a BER>0.2 for the whole investigated range of optical SNR.
